# The differential effects of intimate terrorism and situational couple violence on mental health outcomes among abused Chinese women: a mixed-method study

**DOI:** 10.1186/s12889-015-1649-x

**Published:** 2015-03-31

**Authors:** Agnes Tiwari, Ko Ling Chan, Denise Shuk Ting Cheung, Daniel Yee Tak Fong, Elsie Chau Wai Yan, Debbie Hoi Ming Tang

**Affiliations:** School of Nursing, Li Ka Shing Faculty of Medicine, The University of Hong Kong, 4/F, William M.W. Mong Block , 21 Sassoon Road, Pokfulam, Hong Kong, China; Department of Social Work and Social Administration, The University of Hong Kong, Room 534, Jockey Club Tower, The Centennial Campus, Pok Fu Lam, Hong Kong, China; Po Leung Kuk, 66 Leighton Road, Leigton Hill, Hong Kong, China

**Keywords:** Intimate partner violence, Intimate terrorism, Situational couple violence, Mental health, Chinese women

## Abstract

**Background:**

Distinctions have been made between the two main forms of intimate partner violence: intimate terrorism (IT) and situational couple violence (SCV), depending on whether the violence is part of a general pattern of control. Differential effects also exist between IT and SCV. However, the IT/SCV distinction and their differential effects have yet to be demonstrated in violent intimate relationships in China. We aimed to identify IT and SCV among Chinese women who reported partner violence in Hong Kong and to differentiate the effects of IT and SCV on their mental health outcomes.

**Methods:**

A mixed-method design was used in a cross-sectional study to collect quantitative and qualitative data from women 18 years of age or older who had been victims of intimate partner violence in the past year. Six hundred and thirteen women were recruited from 18 districts in Hong Kong. Quantitative instruments were administered to assess intimate partner violence, control by an intimate partner, and mental health outcomes. Individual face-to-face interviews were conducted with 200 of the women to capture their experiences of intimate partner violence and the context in which it occurred.

**Results:**

Of the 613 women, 215 (35.1%) were identified as victims of IT and 324 (52.9%) as victims of SCV. Compared to SCV victims, IT victims reported significantly more violence-related physical injury (*p* < 0.001), higher use of medical services (*p* < 0.001), and more symptoms of depression (*p* < 0.001) and posttraumatic stress disorder (*p* < 0.001). The interviews revealed two broadly different pictures with IT victims describing their relationship problems as serious and life-threatening, and physical violence was part of the controlling behaviors used by their partners. Such details were not reported by those in the SCV group.

**Conclusion:**

Our findings indicate that violence in intimate relationships in China is not a unitary phenomenon, and it has at least two forms, IT and SCV, which were shown to have differential effects on Chinese women. The findings regarding the IT/SCV distinction and their differential effects on mental health outcomes have implications for policy, research and practice.

**Trial registration:**

Trial registration: ClinicalTrials.gov NCT01206192.

## Background

Intimate partner violence (IPV), defined as “physical violence, sexual violence, stalking, and psychological aggression (including coercive tactics) by a current or former intimate partner” [1], is the most common form of violence against women [2]. It has long been recognized as a public health problem [3] with negative health and social consequences for the victims, their families, and the community [4]. In the past three decades, there has been a growing body of knowledge about the dynamics of IPV [5,6]. In particular, there is increasing acknowledgement that IPV is not a single phenomenon [7,8], and at least two distinct forms have been proposed, namely, intimate terrorism (IT) and situational couple violence (SCV) [9].

Johnson’s typology of domestic violence ^a^ [9] provides the theoretical basis for defining and differentiating IT and SCV. Central to Johnson’s typology is whether the violence is part of a general pattern of control. Specifically, IT is defined as “an attempt to dominate one’s partner and to exert general control over the relationship” (p.323) [10]. Such domination involves the use of a wide range of power and control tactics, including violence. Thus, IT is conceptualized as a matter of control that is rooted in the patriarchal traditions of male dominance in intimate relationships [9]. SCV, however, is defined as “intimate partner violence that is not embedded in a general pattern of controlling behaviors” (p.324) [10]. Conceptualized as a matter of conflict, SCV is rooted in the stresses of family life and that some of the conflict situations may escalate to violence [9]. Although Johnson acknowledges that IPV encompasses physical and sexual violence, stalking and psychological aggression, his typology is based on physical violence only. Thus, in this paper, the conceptualization and measurement of IT and SCV are based on physical violence in intimate relationships, consistent with Johnson’s typology [9].

IT and SCV are thought to differ not only in etiology but also in their effects on the victims [7]. The empirical evidence about the differential effects of IT and SCV on health outcomes has been increasing to date. For example, studies have revealed that, compared to SCV victims, IT victims report more violence-related injuries [10-13], more symptoms of depression and posttraumatic stress disorder (PTSD) [10,14,15], higher psychological distress [12], greater loss of work or activity time [12], and higher rates of substance abuse [14]. Not only are these findings important for understanding the effects of IPV on its victims, the differential effects also have implications for screening and intervention strategies. In particular, practitioners should distinguish between IT and SCV before implementing interventions. While the use of mediation might help couples involved in SCV to resolve conflicts and solve problems, such a strategy can be very dangerous for women victims of IT if they disclose information about the abuse to an outsider in front of their controlling partner.

It is necessary to assess the level of control used by an intimate partner to ascertain whether the violence in an intimate relationship is part of a general pattern of control. Previous studies have used cluster analysis to identify the high level of control that is associated with IT [10-14]. However, using cluster analysis to distinguish a high from a low level of control is problematic for two reasons [9]. First, the nature of the clusters is dependent on the nature of the sample. As such, cluster analysis will find a high control cluster and a low control cluster even in a sample with few or no high-control individuals. Thus, the high control cluster is only relative to the rest of the sample and not necessarily the high level of control that characterizes IT. Second, there is no operational definition of a ‘cluster’ or ‘clusters,’ and without a specified set of criteria for defining clusters, it is not possible to replicate previous clusters in subsequent studies. In light of the limitations of using cluster analysis to differentiate levels of control [9,15], we recently validated a Chinese version of the Revised Controlling Behavior Scale (CBS-R) [16] with a cut-off point to distinguish a high level of control from a low level of control [17]. The present study is the first to use a validated cut-off point, rather than cluster analysis, to distinguish between high and low control in violent intimate relationships.

Additionally, empirical research to date [10-15] has only employed a quantitative approach to make distinctions between IT and SCV. The lack of qualitative information about IT and SCV is a glaring knowledge gap in light of the assumption that IT and SCV are also qualitatively distinct [9]. The present study was designed to enhance our understanding of IT and SCV by collecting quantitative data on the use of violence and control in intimate relationships, as well as eliciting qualitative information about the personal experiences of IPV and the context in which it occurs.

Notwithstanding the empirical evidence relating to IPV typology in general and the IT/SCV distinction, in particular [10-15], the transferability of the previous findings to Chinese intimate relationships cannot be assumed. As yet, no studies have considered the use of control in violent Chinese intimate relationships, nor differentiated the effects of IPV on mental health outcomes in the context of high or low control by an intimate partner (i.e. IT *vs* SCV) [18,19]. It is possible that IT, characterized by male dominance and power over women, prevails in violent Chinese intimate relationships. However, given the effect of globalization and Westernization in recent decades, power dynamics in Chinese intimate relationships could have been altered to the extent that Chinese women are rendered more or less susceptible to IPV [20]. Therefore, it is necessary to examine violence and control in Chinese intimate relationships.

The aim of this study was to adopt a mixed-method design to investigate the use of violence and control in Chinese intimate relationships and to examine the differential effects between IT and SCV on abused Chinese women’s mental health outcomes.

## Methods

This was a mixed-method study with participants recruited from shelter agencies and community centers in all 18 districts in Hong Kong. Agency samples were recruited from shelters providing residential refuge for abused women and Family and Child Protective Services Units (FCPSUs) of the Social Welfare Department of the Hong Kong Special Administrative Region Government. Community samples were recruited from community centers that were operated by non-governmental organizations. The decision to have both agency and community samples was deliberate because the mix of IT/SCV victims is thought to be different between agency and community samples. Johnson’s typology [9] suggests that there are more IT than SCV victims in the agency samples and vice versa in the community samples.

Eligibility criteria are: Chinese women who were 18 years of age or older and screened positive for IPV victimization in the past year. Any woman whose abuser was not her intimate partner was excluded. A sample size calculation was made based on the assumption of a small-to-medium effect size (d = 0.3) [21] of the health effects of IT and SCV. Because the presence of violence and control in intimate relationships had to be identified by statistical analysis, group identity of IT and SCV participants were not known until the data analysis. Based on the results of a previous study [11], the prevalence of IT and SCV cases among all forms of IPV is approximately 22% and 59%, respectively. Hence, the ratio of the sample size of IT to SCV victims was taken to be 1:2.6 in the sample size calculation. G* Power 3 software [22] was used to calculate the required sample size to detect a small-to-medium difference in health effects between the SCV and IT victims. Using a two-tailed *t*-test with the power of .80 and a significance level of .05, the calculation indicated that a sample size of 438 was needed. Given that approximately 80% of all forms of violent relationships consist of SCV and IT [11], and assuming a completion rate of 90% among the participants, the sample size to be recruited was inflated to 609 Chinese women with a past-year history of IPV victimization.

A total of 613 women were recruited for the study and they were all administered the quantitative measures described below. The first 200 women were also qualitatively interviewed individually. The research was conducted in Cantonese which is the dominant dialect in Hong Kong.

### Measurements

#### Quantitative

For the screening process to establish whether or not women were eligible for inclusion in the study, IPV victimization within the past year was assessed by the Chinese version of the Abuse Assessment Screen (C-AAS) [23], which was validated as an appropriate screening instrument for identifying IPV among Chinese women. The C-AAS consists of five dichotomous yes/no items designed to identify Chinese women’s experience of IPV victimization. For the purpose of this study, women who reported a history of physical violence by an intimate partner in the 12 months preceding the study were screened as IPV positive. Also, IT is defined as physical violence by an intimate partner with a high level of control and SCV is physical violence by an intimate partner with a low level of control. The tools used to measure IT and SCV are the Revised Conflict Tactics Scale (for physical violence) and the Revised Controlling Behaviors Scale (for levels of control), as elaborated below.

Physical violence was assessed using the physical assault subscale of the Chinese version of the Revised Conflict Tactics Scale (C-CTS2) that was previously validated with satisfactory validity and reliability [24]. Specifically, the self-reported frequency of physically violent acts perpetrated by the participant against her partner and the frequency of physically violent acts she reported as perpetrated by her partner against her in the past year were recorded. Examples of physically violent acts include: *throw something; beat; push, grab or shove; slap or hit; kick; hit with an object; threaten with a weapon;* and *use a weapon to hurt*. The physical assault subscale consists of eight items that are rated on a 7-point Likert scale, ranging from *0* (*never)* to 6 (*more than 20 times in the past year)*.

The investigators developed questions to assess the *escalation* and *severity* of physical violence. The question about escalation of violence was: “During the past 12 months when you and your partner have been/were together, has the use of physical force increased, stayed the same, or decreased?” Both the use of physical force by the participant against her partner and that used by her partner against her were assessed. Responses were coded as 1 = *increased*, 2 = *stayed the same*, or 3 = *decreased*. The questions about the severity of violence asked the participant to recount, during the past/last year: “How many times were you and/or your partner physically injured?” (e.g., *knocked down, bruised, scratched, cut, chocked, bones broken, eyes or teeth injured*); and “In how many of these fights in which you and/or your partner were physically injured, did you and/or partner go to a doctor, clinic, or hospital for medical treatment?” Responses were rated on a 5-point Likert scale ranging from 0 (*never*) to 4 (*always*).

The use of control by the participant and her partner was assessed using the Chinese version of the Revised Controlling Behaviors Scale (C-CBS-R) [17]. The C-CBS-R consists of 32 items categorized into 7 subscales: economic control (*e.g., control the other one’s money*), threatening control (*e.g., threaten to disclose damaging or embarrassing information*), intimidating control (*e.g., use of nasty looks and gestures to make the other one feel bad or silly*), emotional control (*e.g., call the other unpleasant names*), isolating control (*e.g., check up on the other one’s movement*), using children (*e.g., make the other one feel bad about the children*, and minimizing (*e.g., falsely accuse the other one of using violence*). The frequency of using controlling behaviors was rated on a 5-point Likert scale ranging from 0 (*never*) to 4 (*always*). A mean score of 1.145 was previously validated as the cut-off score to dichotomize high and low levels of control in violent Chinese intimate relationships [17].

Depressive symptoms experienced by the participant in the past two weeks were measured using the Chinese version of the Beck Depression Inventory Version II (C-BDI-II) [25]. The C-BDI-II is a 21-item inventory in which the ratings for each of the items range from 0 (*symptom not present*) to 3 (*symptom strongly present*). The total score, calculated by summing the scores of each item, ranges from 0 to 63, with 0–13 indicating minimal depression, 14–19 indicating mild depression, 20–28 indicating moderate depression, and 29–63 indicating severe depression. The C-BDI-II has been validated and has demonstrated satisfactory internal consistency reliability [26].

PTSD symptoms were assessed using the Chinese version of the PTSD Checklist Civilian Version (C-PCL-C) [27]. The C-PCL-C is a 17-item measure designed to elicit self-reports on three symptom clusters of PTSD, namely, *re-experiencing*, *avoidance*, and *hyper-arousal*. The score of each item ranges from 1 (*not at all*) to 5 (*extremely*) and all 17 items add up to a maximum score of 85. The C-PCL-C was previously validated with satisfactory sensitivity and reliability for identifying PTSD symptoms in the Chinese population, and optimal diagnostic efficiency was demonstrated based on a mixed scoring criteria (i.e., a minimum symptom score of 4 for individual items, and a total score of 50 as the cut-off) [27].

Information about socioeconomic and demographic characteristics was collected to assess and to control for confounding effects on mental health. This information included age, marital status, number of children, employment status, years living in Hong Kong, educational level, financial hardship, and whether financial support was received in the preceding 12 months.

#### Qualitative

The personal experiences of Chinese women regarding IPV were elicited using in-depth, individual interviews. As guided by our previous research [28], we adopted a culturally appropriate, empathetic questioning technique to ensure that the women were comfortable and open enough to be able to share their abusive experience (often considered to be a family shame) with our experienced researchers. As Chinese women have been shown to be more comfortable using narratives and idioms to describe their intimate relationship [28], we began by using a frequently-used Chinese idiom, *Every family has its problems*, and asked the woman to comment on it: e.g., “What do you think about this saying?” As she responded, we encouraged her to elaborate by using prompts such as: “Women often tell us that many of the family problems are related to their relationship with their partner. Can you give us examples of relationship problems that you have had with your partner?*”* We followed up by asking: “We have found that in relationships similar to what you have just described, physical violence is only part of the picture. What else did he say and/or do that made you feel uncomfortable?” To further explore the power dynamics of the relationship, we asked more probing questions with caution and sensitivity, while acknowledging the women’s experiences, for example, “Are you afraid of your partner?” We followed up on a “yes” response with questions like: “Some women have told us that their partners made them do things that they did not want to. Do you have this kind of experience too?” If “yes”, we invited her to elaborate by asking the following: “Tell me an incident when this happened.” “What do you think would happen if you did not do as he said?” “How often do you do as he says?” “Have you thought about not complying with his demand?” “Did you act on it?”“ What happened then?” Finally, to help us understand her perception of the partner, we asked the participant to “Describe your partner to someone like me who does not know him”.

### Ethical consideration, recruitment, and data collection

The study was approved by the Institutional Review Board of the University of Hong Kong/Hospital Authority Hong Kong West Cluster. The study was conducted between September 2010 and September 2012. Participants were recruited by invitations posted in the newsletters, on the boards of the shelters and community centers, and invitations sent to the social workers in the FCPSUs. Our research assistants contacted those women who expressed an interest in participating in the study and arranged a meeting to assess their eligibility. An individual face-to-face interview was used to assess eligibility, which was conducted in a private room provided by the host shelter, FCPSU or community center. During these interviews, our trained research assistants explained to the women the purpose of the study, potential benefits and risks, and the rights of research subjects, before obtaining written informed consent. In addition, an assurance of confidentiality was reiterated, including their participation in the study and information they provided. The potential participants were also reassured that they were able to withdraw from the research at any time with no adverse effects on the services that they were receiving from the institution. The women took as long as necessary to decide whether they wanted to participate.

The C-AAS was administered to those women who provided written consent to screen them for eligibility. Those women who did not meet the inclusion criteria were thanked for their time with the assurance that no further contact would be made. Those women who met the inclusion criteria were assessed for measures of physical assault, controlling behaviors, depressive symptoms, PTSD symptoms, and sociodemographics.

In addition, the first 200 cases also were invited to participate in face-to-face, individual interviews. All but 2 of the interviews were digitally recorded with the women’s permission. Field notes also were kept to document the women’s non-verbal cues, including gestures, facial expressions, emotions, tone of voice, and silence. The average duration of an interview was about 45 minutes.

Upon completion of the quantitative measures and the individual interview, the women were de-briefed and provided community resources for abused women, if necessary, to ensure that participation in the study did not result in adverse effects on their physical or psychological well-being. In addition, in view of the highly sensitive and potentially risky nature of the investigation, there were safeguards in place to ensure the safety of the participants and researchers. For example, researchers were trained to conduct ethical research on violence and the standard of their performance was assessed by the principal investigator. The researchers were only allowed to conduct the recruitment and/or data collection after they had been assessed to be satisfactory by the principal investigator. Also, for the purpose of recruitment, data collection and referral, the researcher ensured that the participant was interviewed alone, in a private room so as to make certain that her male partner would not find out about her disclosure of IPV or use of referral services.

### Statistical analysis

Data were analyzed with the Statistical Package for Social Sciences (SPSS version 20.0, Chicago, Illinois, USA) and the level of significance was set at *p* < .05.

First, the IPV of the participants was categorized into IT and SCV, based on: (1) the physical violence perpetrated by the participant and her partner according to her self-report and her report about her partner (as measured by C-CTS2 physical assault subscale); and (2) the use of coercive control by the participant and her partner, according to her self-report and her report about her partner (as measured by the C-CBS-R). The level of coercive control in the violent intimate relationship was differentiated into high (>1.145) or low (≤1.145) using a score of 1.145 on the C-CBS-R as the cut point [17]. To differentiate between IT and SCV victimization by partner, the following ‘formula’ was adopted: IT victimization = partner’s C-CTS2 score of ≥ 1 + partner’s C-CBS-R >1.145 while SCV victimization = partner’s C-CTS2 score of ≥ 1 + partner’s C-CBS-R ≤1.145.

Next, descriptive statistics were performed to illustrate the sociodemographic composition of the sample and the prevalence of IT and SCV. Chi-square tests and *t*-tests were performed to assess differences between the IT and SCV victims in terms of their demographic characteristics, socioeconomic status, and intimate partner violence experiences, to provide a preliminary comparison of the study variables in the IT and SCV groups.

Multiple logistic regression analysis was performed to identify the risk factors associated with IT, using the demographic and socioeconomic factors as independent variables, after controlling for demographic and socioeconomic factors (age of couples, age difference between the couples, marital status, education, immigrant status, number of children, employment status of the couples, source of recruitment, financial hardship, and financial support received). The results are expressed as crude odds ratios and adjusted odds ratios (AOR) relative to the reference group(i.e., SCV victims).

Linear regressions were conducted to examine the differential effects between IT and SCV on abused Chinese women, that is, whether IT victimization, as compared to SCV victimization, predicted different mental health outcomes (depressive symptoms as measured by C-BDI-II and PTSD symptoms as measured by C-PCL-C). Potential confounding demographic and socioeconomic factors were adjusted in the model.

### Qualitative analysis

Three of the researchers (AT, GL, KL) independently and repeatedly read the transcripts using the processes of intuiting, analyzing, and synthesizing [29]. Key words and phrases were tentatively identified and grouped into categories during the process. Similar categories were clustered together to form themes. The tentative categories and themes were then subjected to critical review and scrutiny during repeated rounds of group discussions, with revisions being made as appropriate. The recursive processes of independent data analyses and group critical scrutiny took place over several months until consensus was reached and a rich description was derived of the contexts in which violence occurred in Chinese intimate relationships.

## Results

### IT and SCV in Chinese intimate relationships

A total of 539 women were classified as IT/SCV victims. Of these women, 215 (39.9%) were IT victims and 324 (60.1%) were SCV victims. Furthermore, 284 (52.7%) were recruited from agency sites (shelters and FCPSUs) and 255 (47.3%) were recruited from community centers.

The male partners of 215 (39.9%) of the women who participated in the study were identified as having perpetrated physical violence, based on the C-CTS2 Physical Assault subscale (mean score = 22.24 ± 30.87), and as having used a high level of control based on the C-CBS-R (mean score = 2.22 ± 0.67;i.e., >1.145). None of these 215 women were found to have perpetrated physical violence against their partner (C-CTS2 mean score = 0.00 ± 0.00) and their use of control in intimate relationship was low (C-CBS-R mean score = 0.26 ± 0.23; i.e., cut-off score ≤1.145). Based on Johnson’s typology of domestic violence [8], these 215 women were classified as IT victims and their partners as IT perpetrators. Most of these women (81.4%) were recruited from the shelters and FCPSUs (the agency sample).

The partners of 324 (60.1%) women were identified as having perpetrated physical violence (C-CTS2 mean score = 4.56 ± 9.11) that was accompanied by a low level of control (C-CBS-R mean score = 0.58 ± 0.28; i.e., ≤1.145). Of the 324 women, 249 (76.9%) were found to have perpetrated no physical violence, and none had used high control in intimate relationships (i.e. C-CBS-R score ≤1.145). Thus, the 324 women were classified as SCV victims and their partners as SCV perpetrators. About twothirds of these women (66.4%) were recruited from the community centers.

Among the remainder of the sample (n = 74), both the women and their partners were found to have perpetrated physical violence and to have used high control that matched the two other types of IPV in the Johnson’s typology [9], Mutual Violent Control (MVC) and Violent Resistance (VR). These cases were not included in the analyses for this paper and they will not be reported here.

### Sociodemographic characteristics

The sociodemographic characteristics of the 539 women that made up the IT/SCV sample are shown in Table 1. Half of the women, but less than 25% of their partners, were aged 20–39. About 26% of the couples had an age difference of more than 10 years, with the male partner older than the woman. Just over twothirds of the women were still married to their partners. While 72% of the partners were in paid employment, more than 70% of the women were not. The majority (95.4%) of the women had children. About onethird of the women had less than 9 years of formal education, and many of them (74.6%) lived in Hong Kong for less than 7 years^b^. About 62% of the women reported experiencing financial hardship in the preceding 12 months, but only 38% received formal financial aid (e.g., from the government).

**Table 1:** **Sociodemographics and abuse characteristics of IT and SCV victims**

	**Total (n = 539, 100%)**		**IT (n = 215, 39.9%)**		**SCV (n = 324, 60.1%)**		***p*** ^a^
	**n**	**%**	**n**	**%**	**n**	**%**	
*Women*							
**Age** (years)							0.530
20-39	284	52.8	114	53.0	170	52.6	
40-49	145	27.0	62	28.8	83	25.7	
≥50	109	20.3	39	18.1	70	21.7	
**Education** (years)							0.605
≤9 years	354	65.7	144	67.0	210	64.8	
>9 years	185	34.3	71	33.0	114	35.2	
**Living in Hong Kong** (years)							0.002*
≥7 years	136	25.4	39	18.2	97	30.1	
<7 years	400	74.6	175	81.8	225	69.9	
**Employment status**							0.001*
Paid employment	160	29.7	46	21.4	114	35.2	
Unemployed	379	70.3	169	78.6	210	64.8	
*Women’s partners*							
**Age**							0.087
20-39	127	23.7	43	20.3	84	25.9	
40-49	179	33.4	66	31.1	113	34.9	
≥50	230	42.9	103	48.6	127	39.2	
**Employment status**							0.002*
Paid employment	382	72.3	134	64.7	248	77.3	
Unemployed	146	27.7	73	35.3	73	22.7	
**Marital status**							<0.001*
Married	365	67.8	104	48.6	261	80.6	
Divorced/separated	173	32.2	110	51.4	63	19.4	
**Age difference between woman and her partner** (years)							<0.001*
≤10	390	74.1	128	61.5	262	82.4	
>10	136	25.9	80	38.5	56	17.6	
**Number of children**							0.628
None	25	4.6	11	5.1	14	4.3	
1	194	36.0	83	38.6	111	34.3	
2	228	42.3	84	39.1	144	44.4	
≥3	92	17.1	37	17.2	55	17.0	
**Place of subject recruitment**							<0.001*
Shelters	177	32.8	110	51.2	67	20.7	
FCPSUs	107	19.9	65	30.2	42	13.0	
Community centers	255	47.3	40	18.6	215	66.4	
**Financial hardship**							<0.001*
Yes	334	62.1	170	79.4	164	50.6	
No	204	37.9	44	20.6	160	49.4	
**Financial support**							<0.001*
Yes	205	38.1	104	48.6	101	31.2	
No	333	61.9	110	51.4	223	68.8	
**Afraid of the partner**							<0.001*
Yes	301	55.8	183	85.1	118	36.4	
No	238	44.2	32	14.9	206	63.6	
**Duration of IPV** (years) (Mean **±** SD)	5.55 ± 6.54		4.51 ± 5.47		6.48 ± 7.49		<0.001*
**Escalation of violence**							<0.001*
Yes, increasing	182	38.3	129	60.8	53	20.2	
Stayed the same	174	36.6	43	20.3	131	49.8	
No, decreasing	119	5.1	40	18.9	79	30.0	
**Physical injury** (number of times)							
Woman (Mean **±** SD)	1.38 ± 3.31		2.67 ± 4.61		0.52 ± 1.50		<0.001*
Partner (Mean **±** SD)	0.04 ± 0.30		0.02 ± 0.15		0.06 ± 0.37		0.123
**Injury resulting in the use of medical services** (number of times)							
Woman (Mean **±** SD)	0.38 ± 1.23		0.75 ± 1.78		0.14 ± 0.51		<0.001*
Partner (Mean **±** SD)	0.02 ± 0.16		0.01 ± 0.12		0.02 ± 0.18		0.588

^a^ *p*-value obtained by chi-square test and independent *t*-test.

*p-value <0.05.

Significant differences were found between the IT and SCV victims in terms of their sociodemographic characteristics, including: place of recruitment, employment status, years living in Hong Kong, marital status, couple’s age difference, financial hardship, and financial support received (Table 1). Compared with SCV victims, significantly more IT victims were recruited from agencies (shelters and FCPSUs), had no paid employment, lived in Hong Kong for less than 7 years, were divorced or separated from their partner, had an age difference with their partner of more than 10 years, experienced financial hardship, and received financial support in the preceding year.

Furthermore, the duration of IPV was significantly longer for SCV (mean = 6.48 years) than IT (mean = 4.51 years). Also, IT victims reported a significant increase in their partner’s use of violence over time, more IPV-related physical injury, and higher use of medical services, compared with SCV victims.

### Factors associated with IT victimization

A series of logistic regression analyses were conducted to uncover the factors associated with IT victimization by comparing IT victims to SCV victims. As shown in Table 2, the likelihood of IT victimization was lower among women with a couple’s age difference of ≤ 10 years (AOR = 0.383). However, the chance of experiencing IT victimization was higher among women who were divorced or separated (AOR = 1.677), were experiencing financial hardship in the preceding year (AOR = 2.161), or were from the agency samples (AOR = 4.915). The *p*-value of the Hosmer-Lemeshow test was 0.585 and the Nagelkerke R Square was 0.341, indicating goodness of fit of the model.

**Table 2:** **Adjusted** ^a^ **analysis of factors associated with IT in comparison with SCV victims (n = 539)** ^b^

**Variables**	**Crude OR**	**95% CI**	**Adjusted OR**	**95% CI**
Age (with reference to ≥50)				
Women				
20-39	0.831	(0.526,1.313)	0.498	(0.202,1.227)
40-49	0.746	(0.447,1.244)	0.800	(0.372,1.722)
Partner				
20-39	1.584	(1.01,2.485)	1.297	(0.536,3.135)
40-49	1.389	(0.931,2.071)	1.402	(0.725,2.712)
Age difference of ≤ 10 years between woman and her partner	2.924**	(1.957,4.369)	0.383**	(0.197,0.743)
Divorced/separated	0.228**	(0.155,0.335)	1.677*	(1.018,2.764)
Number of children (with reference to 0)				
1	1.168	(0.478,2.852)	0.408	(0.136,1.224)
2	1.347	(0.585,3.102)	0.441	(0.145,1.338)
≥3	1.051	(0.454,2.432)	0.505	(0.153,1.666)
Education ≤ 9 years	0.908	(0.631,1.308)	0.954	(0.598,1.521)
Living in Hong Kong < 7 years	0.517*	(0.339,0.787)	1.015	(0.568,1.814)
Employment				
Women: unemployed	0.501**	(0.337,0.746)	1.246	(0.752,2.063)
Partner: unemployed	0.540*	(0.367,0.795)	0.857	(0.501,1.467)
Financial hardship	0.265**	(0.178,0.394)	2.161**	(1.286,3.633)
Financial support	0.479**	(0.335,0.684)	1.131	(0.698,1.831)
Recruited from shelter/FCPSUs	0.249**	(0.17,0.364)	4.915**	(2.905,8.317)

^a^Adjusted by age of couples, age difference between the couples, marital status, education, immigrant status, number of children, employment status of the couples, source of recruitment, whether the women had experienced financial hardship, and whether the women had received financial support in the previous year.

^b^Comparing IT and SCV victims only.

*p < 0.05, **p < 0.001.

### Differential effects of IT and SCV on victims’ mental health

Linear regression demonstrated that compared to SCV victimization, IT victimization predicted statistically higher depressive symptoms [as shown by BDI scores] (beta = 16.8, p < 0.001) and PTSD symptoms [as shown by PCL scores] (beta = 22.4, p < 0.001), after controlling for demographic and socioeconomic factors as listed in Table 3.

**Table 3:** **Linear regression examining the influence of IT victimization, compared to SCV victimization, on mental health outcomes**

**Measure and scale**	**n**	**Unadjusted analysis**			**Adjusted** ^a^ **analysis**		
		**β**	**95% CI**	**p-value**	**β**	**95% CI**	**p-value**
Depression and C-BDI-II	539	18.4	(15.9,21.0)	<0.001	16.8	(14.0,19.7)	<0.001
PTSD and C-PCL-C	539	26.4	(23.1, 29.6)	<0.001	22.4	(18.8,26.1)	<0.001

^a^Adjusted by age of couples, age difference between the couples, marital status, education, immigrant status, number of children, employment status of the couples, source of recruitment, whether the women had experienced financial hardship, whether the women had received financial support in the previous year.

Table 4 shows a more detailed breakdown of depressive and PTSD symptoms among IT and SCV victims. Specifically, the percentage of IT victims who reported severe depressive symptoms was significantly higher than that of SCV victims (59.9% *vs* 15.7%). Similarly, the percentage of IT victims with C-PCL-C scores indicative of PTSD was significantly higher than that of SCV victims (65.6% *vs* 19.1%).

**Table 4:** **IT/SCV victims reporting depressive/PTSD symptoms**

**Characteristics**		**IT**		**SCV**		***p*** ^a^
		**(n = 215)**		**(n = 324)**		
		**n**	**%**	**n**	**%**	
C-BDI-II scores	Minimal (0–13)	31	14.4%	197	60.8%	<0.001
	Mild (14–19)	20	9.3%	31	9.6%	
	Moderate (20–28)	36	16.7%	45	13.9%	
	Severe (29–63)	128	59.5%	51	15.7%	
C-PCL-C scores	<50	74	34.4%	262	80.9%	<0.001
	≥50	141	65.6%	62	19.1%	

^a^p-values obtained by *t*-test.

### Personal experiences of IPV

The first 200 of the 613 participants were interviewed. The women vividly described their personal experiences of IPV in the individual face-to-face interviews. They gave detailed illustrations of their partners’ violent behaviors, not only about the acts themselves but also about the surrounding circumstances. Analysis of the women’s accounts revealed two broadly different pictures of IPV, as illustrated in the following themes:

#### Theme 1: Relationship problems with partners

In one group of women (n = 91), their experiences of IPV were serious and life-threatening, as these two women recounted:

*He beat me up with an iron rod over and over again in the dark alley behind his shop…I thought I was going to die…if not for the person who happened to come in to dump the rubbish…* (#008): age 34, partner aged 48; mother of a 5-year old daughter; a university graduate. Her injuries from IPV included a bite to her breast requiring suturing. In the interview, she also described how her partner(a director of a listed company) controlled where she went (in a chauffeur-driven car), what she did (accompanied by two maids), and how much she spent (she had no credit cards and only the equivalent of US$100 in her bank account).

*The worst time was when we were in China, he tried to kill me so many times…I was lucky to be alive…I could not get help from anyone there…because wife beating was so much part of life…there is more protection for people like me in HK but I shall never be free from him.* (#127): age 64, partner aged 69; mother of 5 grown children. She was abused by her partner throughout 44 years of marriage. She moved from China 33 years ago. When completing the C-CBS-R, she initially had a problem responding to many of the items because she thought that her husband had a right to control the family income, punish her, and decide what she was allowed to do, including stopping contact with her parents and siblings.

For the remaining women (n = 109), none of them reported their IPV experiences as life-threatening, rather, relationships with their partner were described as frustrating with recurrent arguments, as told by these two women:

*Living with in-laws was a huge problem. I just wanted his father to be more hygienic and he didn’t like it. Every time I raised the issue, he would become very angry and even slapped me in the face.* (#089): age 36, partner aged 51; mother of a 7-year old son. The IPV started a year ago when she came from China. She blamed poor living conditions and conflicts with in-laws for “*ruining*” her relationship with her partner.

*We were fine until he lost his job and started to gamble on the horses. He got angry easily and swore all the time. If I told him not to swear in front of the kids, he would get worse…and often ended up hitting me and even the kids.* (#011): age 39, partner aged 54; mother of two children. She had a three-year history of IPV, with an unemployed partner who was reported to have a problem controlling his temper.

#### Theme 2: Context in which violence occurred in intimate relationships

For the group of women (n = 91) who described their intimate relationship problems as serious and life threatening, violence was part of the controlling behaviors used by the partner to make them behave as they were told, as these women said:

*I was not allowed to work because he thought I would become too clever for my own good. Violence and starvation were common…to instil fear in me.* (#031): age 30, partner’s age 39. She is an emaciated mother of a toddler whom she had to leave behind while fleeing from her violent partner. She was sponsored by her partner to come to Hong Kong and she was still waiting for a decision on her application for right of abode. In the interview, she broke down several times when talking about how she was torn between going back (and face further violence and starvation) and leaving him (but risk deportation).

*Violence is not the worst part of our relationship…what is really frightening is not knowing what would upset him and when. I mean I have to watch every sign that says he is not happy and that something bad is going to happen.* (#030): age 58, partner aged 78; mother of two grown sons. At the age of 26 her mother married her to her present partner (20 years her senior) in return for money, and she had a history of more than 30 years of IPV. She revealed in the interview that she was only allowed to go to the shops and market near her home since coming to HK more than 10 years ago, and that coming to the shelter was the first time for her to travel outside the district where she lived.

The accounts provided by these 91 women are consistent with a general pattern of power and control that characterizes IT [9]. On the contrary, for the women who described their relationship problems as frustrating with recurrent arguments (n = 109), their accounts hardly made any reference to the use of controlling behaviors by their partners. Indeed, they talked about how they actively tried to resolve the recurrent problems in their relationship, even though their attempts did not always pay off, as described by these women:

*He was addicted to gambling…and the more he lost, the more he gambled. He borrowed money from friends in my name…and from the loan shark too. What I earned from my part-time job was not enough to repay his debts…and trying to talk some sense into him often ended in more argument or worse…* (#059): age 40, partner aged 55; mother of an 11 year-old son from her previous marriage. She has been cohabiting with present partner for one year, who has threatened to kill her, her son, and himself in a recent violent episode.

*After he lost his job, I came to HK to support him. His family looked down on him and me…Well, instead of showing some appreciation for what I gave up for him, he blamed me for everything. This time, he even hit me and I said to myself, this is it, I am getting out.* (#063): age 31, partner aged 44; no children. She has a 3-year history of IPV. She was a beauty consultant who was getting increasingly frustrated about her partner’s lack of motivation to look for a job.

The personal experiences of IPV as described by these 109 women are consistent with Johnson’s [30] “situationally provoked violence” that characterizes SCV.

## Discussion

To the best of our knowledge, this is the first study to investigate violence and control in Chinese intimate relationships using a mixed-method quantitative-qualitative approach. It is also the first to report on the differential effects between IT and SCV on Chinese women’s mental health outcomes.

About onethird of the women in this study were IT victims who experienced IPV that was accompanied by high control, while about half of the women were SCV victims whose experience of violence by an intimate partner was not accompanied by high control. The uncovering of the two types of IPV in our sample provides empirical support that IPV in Chinese relationships is not a single phenomenon and this is consistent with the Western literature that multiple forms of IPV exist [7,9,31,32]. Furthermore, the revelation that violence may be part of a pattern of control by the partner reinforces the need to assess the context in which it occurs. The findings have implications for measurement of IPV in Chinese relationships. Thus far, studies on IPV in Chinese relationships have focused only on violent acts [18,19]. Future studies should consider assessing the violence as well as the control used by both partners in intimate relationships.

In addition to making distinctions between IT and SCV, we also found that the two forms of IPV have differential effects on Chinese women’s mental health outcomes. Specifically, our IT victims reported more IPV-related injuries, higher use of medical services, and higher depressive and PTSD symptoms, compared with the SCV victims. The women’s reports were consistent with those of earlier studies involving non-Chinese women [10-15]. The findings of the differential mental health effects among abused Chinese women, perhaps, are not surprising. In an earlier review of IPV studies involving Chinese women, we hypothesized that in a shame-oriented Chinese culture, the power and control associated with IPV could have weakened Chinese women’s perception of self and induced shameful feelings that led to the reported adverse mental health outcomes [33]. As the studies reviewed did not include the partner’s use of control in violent relationships, we were unable to find evidence for or against our conjecture. The present study has advanced our understanding of the effect of IPV on mental health of Chinese women by showing that violence that is controlling (that is, IT) has more detrimental effects. These differential effects on mental health outcomes have practical implications. First, it is important that IPV assessment should clearly identify IT victims in order to detect women who are at high risk of serious injury or more deleterious health outcomes. This would allow the implementation of effective interventions to protect the women from future harm and preventable injuries. Second, although victims of non-controlling partner violence (i.e., SCV) may be at relatively lower risk for injury and adverse health outcomes, it should not be assumed that the violence is less dangerous or harmful for these women [32]. Indeed, not only should SCV victims be referred to the appropriate services depending on the causes of the situationally provoked violence, it is also important to remind them that IPV is potentially harmful or even lethal.

The qualitative data elicited from the women have revealed nuances of violent Chinese relationships that quantitative measurements are unable to capture comprehensively. Specifically, through the women’s descriptions, we learn about the fear and suffering endured by women in violent controlling relationships, and the feeling of powerlessness in a society that condones violence against women. The women’s accounts have implications for policy, research and practice. In terms of policy, zero tolerance to any form of IPV [34] should drive social change in Chinese societies that condone violence against women. In terms of research, qualitative research should be conducted with women experiencing controlling violence by an intimate partner to better understand the social context that affects their experience of violence and control, and what interventions would be most helpful for them. In terms of practice, both health and social services providers need to develop unique interventions according to whether IPVis accompanied by high or low control.

Concerns have previously been expressed about the use of cluster analysis to differentiate high and low level of control in violent intimate relationships [9]. In the current study, we used a cut-off score on the C-CBS-R to distinguish between high and low control which, in turn allowed us to make distinctions between controlling and non-controlling violence in Chinese relationships. The C-CBS-R cut-off score show promise as a statistical criterion for identifying low and high control, and has the potential to assist frontline workers to make preliminary distinctions among the different forms of IPV presented by the victims and refer them to the appropriate services.

Among the risk factors of IT identified in the present study, financial hardship is consistent with the finding of an earlier study [32], in which low income was identified as a risk factor associated with IT. In addition, we also found that women who were separated or divorced were at higher risk for IT. It has long been recognized that separation/divorce renders women at high risk of lethality [35].

### Limitations

This study has several limitations. First, the sample may be biased due to self-selection of the participants, particularly those who sought services among the agency samples. Thus, the findings may not be generalizable to those not seeking or receiving services for abused women. Second, the cross-sectional design does not permit us to assess if the two forms of IPV detected are truly different categories or different phases of a violent relationship. Longitudinal studies should be conducted to understand the life course of IPV and to track the development of violence and control in intimate relationships over time. Third, women without physical partner violence were not included in our study, so we were unable to consider the effect of high relationship control in the absence of violence. This is a limitation of Johnson’s IT/SCV typology [9]. This limitation is salient, as an earlier study has shown that a high level of control by an intimate partner is associated with negative health outcomes, even when control does not co-occur with violence [15]. The inclusion of women who experience high level of control by an intimate partner, but not the physical form of IPV, in future studies may help to expand the scope of Johnson’s typology. Finally, while self-reports, like those used in this study, may reveal the kind of IPV that has not come to the notice of the authorities (e.g., the police), self-reports are subject to social desirability and recall bias. Future studies should consider triangulating self-reports with clinical records, such as police reports, and health and social services records.

## Conclusion

The findings in this study indicate that IPV in Chinese relationships is not a single phenomenon, and that two forms of violence, IT and SCV, can be identified based on an assessment of physical violence in the context of level of control. IT and SCV have differential effects on Chinese women, with those experiencing IT reporting more negative mental health outcomes. The use of a mixed-method approach has demonstrated utility in enhancing the breadth and depth of the phenomenon of IPV in Chinese relationships, with the quantitative measures making a distinction between controlling violence (IT) and non-controlling violence (SCV), while the qualitative accounts illustrate the nuances of the context surrounding violent Chinese intimate relationships. The results of the present study have implications for policy, research and practice.

### Endnotes

^a^Johnson adopts ‘domestic violence’ to denote intimate partner violence in his typology.

^b^Application for permanent citizenship is only considered after living in Hong Kong for at least 7 years.
